# Is cognitive conflict really effortful? Conflict priming and shielding effects on cardiac response

**DOI:** 10.1111/psyp.14169

**Published:** 2022-09-08

**Authors:** Yann S. Bouzidi, Guido H. E. Gendolla

**Affiliations:** ^1^ Geneva Motivation Lab, FPSE, Section of Psychology University of Geneva Geneva Switzerland

**Keywords:** action shielding, cardiovascular reactivity, cognitive conflict, effort, pre‐ejection period

## Abstract

Two experiments with *N* = 221 university students investigated the impact of primed cognitive conflict on effort assessed as cardiac response in tasks that were not conflict‐related themselves. Manifest cognitive conflict in cognitive control tasks is confounded with objective response difficulty (e.g., in incongruent Stroop task trials). This makes conclusions about the effortfulness of cognitive conflict itself difficult. We bypassed this problem by administrating pictures of congruent versus incongruent Stroop task stimuli as conflict primes. As predicted, primed cognitive conflict increased cardiac pre‐ejection period (PEP) responses in an easy attention task in Experiment 1. Accordingly, cognitive conflict itself is indeed effortful. This effect was replicated in an easy short‐term memory task in Experiment 2. Moreover, as further predicted, the primed cognitive conflict effect on PEP reactivity disappeared when participants could personally choose task characteristics. This latter effect corresponds to other recent evidence showing that personal action choice shields against incidental affective influences on action execution and especially on effort‐related cardiovascular response.

## Abstract 2

Cognitive conflict is said to be effortful, but conclusive evidence is lacking. One problem is that conflict in cognitive control tasks is confounded with response difficulty. We bypassed this problem by administrating pictures of congruent vs incongruent Stroop task stimuli as conflict primes. Primed conflict indeed increased responses of cardiac pre‐ejection period in tasks that were neither difficult nor conflict‐related themselves. Furthermore, participants’ personal choice of task characteristics could eliminate the conflict effect on effort.

## 1 INTRODUCTION

Cognitive conflict occurs when two incompatible response tendencies exist in one situation (van Steenbergen & Band, 2013). It arises on different levels and in different forms (Inzlicht et al., 2015). High‐order conflict occurs in case of simultaneously activated incompatible action goals and temptations, like wanting to prepare an exam and hanging out with friends at the same time (Duckworth et al., 2019). Low‐order cognitive conflict can appear from the perceptual environment, as in experimental procedures like flanker (Eriksen & Eriksen, 1974) or Stroop (1935) tasks. In both cases, conflict resolution calls for action control engaging executive functions and thus effort (Dreisbach & Fischer, 2012a). On the neural level, conflict‐driven cognitive control is associated with an activation of the anterior cingulate cortex (ACC) (Fröber et al., 2017; van Steenbergen et al., 2012). This brain area is involved in both conflict detection (Botvinick et al., 2001; Botvinick & Braver, 2015) and cost/benefits analyses related to effort exertion (Shenhav et al., 2013; for an integrative view of these two processes in the ACC see Botvinick, 2007) and has been linked to cardiovascular indices of resource mobilization (Silvestrini, 2017; Silvestrini et al., 2022).

We investigated the effect of cognitive conflict on cardiac response to assess effort. Several authors have posited that cognitive conflict is effortful (e.g., Dignath et al., 2020; Dreisbach & Fischer, 2012a; Inzlicht et al., 2015; Kool et al., 2013; Kool & Botvinick, 2018; van Steenbergen, 2015), but surprisingly there is little empirical support for this idea. We think that this lack of conclusive evidence could be caused by the usual strategy to study cognitive conflict with cognitive control tasks—that is, tasks in which correct responses are hardly possible on the automatic level and thus require effortful cognitive control. A perfect example are incongruent trials in a Stroop task (e.g., responding “blue” when the word “red” is written in blue). Accurate responding in such trials requires effortful cognitive control to inhibit a dominant response tendency—automatically reading the conflicting semantic meaning of the color word and responding accordingly. That is, correct responses are *objectivel*y more difficult in incongruent than in congruent trials—and in general, difficult actions require more effort than easy actions (e.g., Richter et al., 2008; Wright et al., 1986). That is, in cognitive control tasks conflict is confounded with objective response difficulty.

The higher objective response difficulty in incongruent cognitive control task trials is not the only reason why cognitive conflict should be effortful. Several conflict researchers have posited that cognitive conflict is aversive and that negative affect is inherent to any type of conflict (Berger et al., 2020; Dreisbach & Fischer, 2012a, 2012b; Inzlicht et al., 2015; Pourtois et al., 2020; Silvestrini & Gendolla, 2019; van Steenbergen et al., 2010). This conflict triggered negative affect is short lived and disappears rapidly—at least in the context of low‐order cognitive conflict (Fritz & Dreisbach, 2015). Conflict is also described as producing feelings like anxiety and frustration (Inzlicht et al., 2015)—though we think that this should actually be reserved for high‐order conflict. However, there is ample evidence that diffuse negative affect, fear, and sadness are associated with low coping potential.^1^ For example, both experienced and implicit sadness and fear increase *subjective* task difficulty and thus effort‐related physiological responses—as long as success is possible and justified (see Gendolla, 2012; Gendolla & Brinkmann, 2005; Gendolla, Brinkmann, et al., 2012, for reviews). Complying with this idea, studies have shown that positive affect can reduce the efficiency of conflict resolution (Berger et al., 2019; van Steenbergen et al., 2009, 2010, 2012). In other words, negative affect seems to be the sine qua non condition that triggers individuals' adaptation to cognitive conflict (for reviews see Dignath et al., 2020; Dreisbach & Fischer, 2015; van Steenbergen, 2015). However, testing whether cognitive conflict is effortful because of its aversiveness effect on subjective demand calls for disentangling the conflict effect from objective response difficulty. Consequently, we applied a *primed cognitive conflict* paradigm that was originally developed by Dreisbach and colleagues (e.g., Dreisbach & Fischer, 2012a, 2015; Fritz & Dreisbach, 2013, 2015) and tested whether cognitive conflict is effortful when response difficulty is objectively low.

### 1.1 Is cognitive conflict itself really effortful?

Effort is defined as the mobilization of resources to carry out instrumental behavior (Gendolla & Wright, 2009). Therefore, effort should be reflected by physiological processes involved in engagement—or in other terms, in active coping—and thus the activity of the sympathetic nervous system (SNS), which is responsible for behavioral activation (Obrist, 1976, 1981). Some studies have quantified effort in terms of pupil dilation (e.g., van der Wel & van Steenbergen, 2018; van Steenbergen & Band, 2013) and found an increased pupil size after incongruent Simon task trials as compared to congruent trials. Nevertheless, the authors of these studies acknowledge that, even though related to SNS activity, pupil dilation may reflect several processes at once (e.g., emotion processes), thus limiting the clarity of a direct link between pupil size changes and effort.

In the tradition of Obrist's (1981) active coping approach and its elaboration by Wright (1996), measures of SNS impact on the cardiovascular system—the body's resource transport system—should be more conclusive. Following this logic, Kuipers et al. (2017) tried to find a link between cardiac contractile force assessed as changes in RZ‐intervals, a measure of sympathetic impact on the heart, during single trials of a cognitive conflict task. Unfortunately, this promising study's results suffered from a temporal overlap between heart rate and RZ‐interval changes, making an interpretation of the found effects difficult: the observed shortened RZ‐intervals following incongruent task trials could have been caused by cardiac preload effects rather than SNS‐based effects on cardiac contractile force. Another study (Spruit et al., 2018) found increased cardiac activity following errors in a cognitive control task. Interestingly, as stated above, both error and conflict detection mechanisms involve the same cerebral area—the ACC (Botvinick et al., 2001; Botvinick & Braver, 2015; Shenhav et al., 2013; Silvestrini, 2017), which is compatible with the idea of a common error/conflict detection mechanism (see Proulx et al., 2012, for an integrative review).

Summing up, measures of SNS impact on the heart appear to be a promising way for testing whether cognitive conflict is indeed effortful, but conclusive evidence is still lacking. We aimed at contributing to fill this gap by disentangling conflict effects from objective response difficulty effects.

### 1.2 Manifest and primed cognitive conflict

To test if cognitive conflict is indeed effortful, most researchers have contrasted SNS activation in incongruent (i.e., conflict‐related) versus congruent (i.e., non‐conflict‐related) task trials. This approach mirrors the usual procedures of studies assessing only performance but not effort. Unfortunately, this means the comparison of trials with different levels of objective difficulty—correct responses in incongruent trials are objectively harder than in congruent trials. Considering the vast evidence of task difficulty effects on effort‐related cardiovascular responses (see Gendolla et al., 2019; Gendolla, Wright, et al., 2012; Richter et al., 2016; Wright & Kirby, 2001, for overviews) from research in the context of motivational intensity theory (Brehm & Self, 1989), this bears a severe problem: it creates a confound.

Finding that correct responses in incongruent task are associated with stronger cardiovascular responses would not clarify whether this occurs because of higher response difficulty or because of conflict‐triggered negative affect. Therefore, building on a research procedure developed by Dreisbach and Fischer (2012a), we primed cognitive conflict instead of inducing manifest response conflict. We used cognitive conflict primes to activate individuals' mental representations of a cognitive conflict during the performance on cognitive tasks that were neither difficult nor conflict‐related themselves. This procedure prevents the confound between conflict and objective response difficulty and can thus test the effortfulness of cognitive conflict itself, according to the conflict‐triggered negative affect hypothesis (Dignath et al., 2020; Proulx et al., 2012).

Summing up, we agree that conflict is aversive, and that low order cognitive conflict should elicit diffuse—or even implicit—negative affect that is associated with difficulty and low coping potential (Silvestrini & Gendolla, 2019). Consequently, cognitive conflict should be effortful.

### 1.3 Effort and cardiac response

We quantified effort based on Wright's (1996) integration of motivational intensity theory (Brehm & Self, 1989) with Obrist's (1976, 1981) active coping approach. Accordingly, especially β‐adrenergic SNS impact on the cardiovascular system is proportional to task demand as long as success is possible and justified. This approach has received ample and consistent empirical support (see Gendolla et al., 2019; Gendolla, Wright, et al., 2012; Richter et al., 2016; Wright & Kirby, 2001, for overviews). The most reliable noninvasive β‐adrenergic impact measure is the cardiac pre‐ejection period (PEP). It reflects cardiac contractile force and is assessed as the time interval (in ms) between the beginning of the left ventricular depolarization and the opening of the aortic valve (Berntson et al., 2004). That is, with increasing cardiac contractility, PEP becomes shorter. Nonetheless, PEP should always be assessed together with heart rate (HR) and blood pressure to monitor eventual preload (ventricular filling) and afterload (arterial pressure) effects. Moreover, increases in SBP, HR, and (to a lower degree) DBP can also index beta‐adrenergic impact on the heart to the extent that blood pressure is determined by myocardial contractility and that HR relies on sympathetic activation rather than parasympathetic withdrawal (Levick, 2010). Taken together, PEP is the most reliable and thus the most suitable cardiovascular index of effort (Kelsey, 2012; Richter et al., 2008).

### 1.4 The present studies

To avoid a confound between cognitive conflict and objective response difficulty effects on effort, we tested the impact of primed rather than manifest cognitive response conflict on cardiovascular response, especially PEP, in the context of objectively relatively easy tasks. Inspired by Dreisbach and Fischer's (2012a) procedure, we used pictures of congruent versus incongruent Stroop (1935) task trials as primes to activate participants' mental representation of cognitive conflict in tasks that were neither difficult nor conflict‐related themselves: an attention task in Experiment 1 and a short‐term memory task in Experiment 2—both in between‐persons designs in which we administered either congruent or incongruent Stroop primes. The between‐persons designs were necessary, because reliable PEP measures require the ensemble averaging of impedance cardiogram signals of at least 20 cardiac cycles (Kelsey et al., 1998; Kelsey & Guethlein, 1990), which is not possible on a trial basis in within‐persons designs. In Experiment 1, we predicted a conflict prime effect on cardiovascular response. Specifically, individuals exposed to conflict primes should show stronger PEP responses than those in the non‐conflict primes condition. To assure that participants really associated incongruent Stroop trials with cognitive conflict, all participants performed a short vocal Stroop task at the beginning of the procedure, as in Dreisbach and Fischer's (2012a) studies.

Experiment 2 aimed at replicating the conflict prime effect on PEP and further tested whether it could be moderated by the personal choice of task characteristics. We based our hypothesis on recent findings showing that giving individuals the possibility to choose among task characteristics fosters commitment (Bouzidi et al., 2022) and shields against incidental affective influences (Falk et al., 2022a, 2022b; Gendolla et al., 2021). As outlined above, cognitive conflict is aversive and thus represents an affective stimulation. Following the logic of an action‐shielding model (Gendolla et al., 2021), we thus predicted that individuals who could personally choose task characteristics should be immunized against primed cognitive conflict's effect on effort. By contrast, individuals to whom the task characteristics were assigned should show the conflict‐related extra effort effect.

The experimenters of the present two studies were hired and unaware of both the experimental conditions and the hypotheses. The data and data coding for the here reported studies are available on Yareta—the open access data archiving server of the University of Geneva: https://doi.org/10.26037/yareta:zvnvr2ajz5fvrlaqoge5pqvl3e

## 2 EXPERIMENT 1

Following Dreisbach and Fischer (2012a), participants first performed a short vocal Stroop (1935) task to give them the experience that Stroop trials could be either conflictual (incongruent trials) or not (congruent trials). Next, all participants performed an objectively relatively easy d2 attention task (Brinkenkamp, 1981), which was not conflict‐related itself. Depending on the between‐person condition, trials started with pictures of either congruent or incongruent Stroop task trials as primes to activate participants' mental representation of conflict or non‐conflict. We expected stronger cardiovascular responses (especially PEP) in the incongruent Stroop prime condition.

### 2.1 Method

#### 2.1.1 Participants and design

For this first test of conflict primes' effect on cardiovascular response we wanted to get valid data of at least 30 participants per condition. We did so, because previous research manipulating either explicit or implicit affect found significant effects of medium size on cardiac reactivity with samples between 20 and 31 participants per condition (e.g., Falk et al., 2022a, 2022b; Framorando & Gendolla, 2018; Gendolla et al., 2021). To compensate for possible data loss because of technical problems with the vocal Stroop task or the physiological measures, we recruited *N* = 90 University of Geneva students with different majors (average age 24 years) with distributed flyers. Participation was compensated with either 10 Swiss Francs (approximately 11 USD) or partial course credit if respondents were first year psychology bachelor students. We asked recruited participants not to drink caffeine nor eat heavy meals 2 hours before their experimental session. Participants were randomly assigned to one of the two Stroop Prime conditions: Incongruent‐Prime versus Congruent‐Prime. The gender distributions were balanced in the two conditions (Incongruent Primes: 31 women, 15 men; Congruent Primes: 30 women, 14 men). Four participants were excluded from the analyses as their PEP and SBP reactivity exceeded the respective condition mean for more than 3 *SD*s. Two participants were excluded because they made about 50% wrong responses in the relatively easy task, suggesting that they did not follow the instructions, leaving *N* = 84 participants. Because of technical problems with the ICG assessment of 4 participants, the final sample for PEP and HR analyses was *N* = 80. A sensitivity analysis run with G*Power (Faul et al., 2007) revealed that our sample size was big enough to detect effects of medium size in our 2 conditions between‐persons design (*α* = .05, power 80%). There were no significant differences between the conditions regarding age, gender, or body mass index (*p*s > .539, see Table S1 in the Supporting Information).

#### 2.1.2 Apparatus and physiological measurement

Cardiovascular measures were taken during habituation (8 min) and task performance (5 min) periods and automatically stored on computer disk. HR (in beats per minute [bpm]) and PEP (in milliseconds [ms]) were continuously measured with a Cardioscreen 2000 hemodynamic‐monitoring system (medis; Ilmenau, Germany). The device noninvasively assessed electrocardiogram (ECG) and impedance cardiogram (ICG) signals with two pairs of spot electrodes (Ag/AgCl; medis, Ilmenau, Germany) placed on the left side of the base of participants' neck and middle axillary line at the height of the xiphoid. Signals were amplified, digitalized (1000 Hz sampling rate), and examined offline with BlueBox 2.V1.22 software (Richter, 2010) applying a 50 Hz low pass filter. ECG R‐peaks were determined using a threshold peak‐detection algorithm and visually confirmed. We calculated the first derivative of the change in thoracic impedance and ensemble averaged 1‐min intervals of the resulting dZ/dt‐signal (Kelsey et al., 1998; Kelsey & Guethlein, 1990). B‐point location was estimated based on the RZ interval of valid heart beat cycles (Lozano et al., 2007), visually checked, and manually corrected if necessary (Sherwood et al., 1990). Following Berntson et al. (2004), PEP was quantified as the time interval between R onset and the B‐point. IBIs were used to determine HR using the same software.

SBP and DBP (in millimeters of mercury [mmHg]) were oscillometrically assessed with a Dinamap ProCare monitor (GeE Healthcare; Milwaukee, WI). We placed a blood pressure cuff over the brachial artery above the elbow of participants' non‐dominant arm. The cuff automatically inflated in 1‐min intervals and blood pressure values were automatically stored. Researchers who are interested in more detailed hemodynamic responses that were unrelated to our hypotheses, can find analyses of cardiac output and total peripheral resistance in the here reported studies in the Supporting Information.

#### 2.1.3 Procedure

Both here reported studies' procedures had been approved by the ethics commission of the University of Geneva. Participants were invited to individual 30 min sessions. The procedure was run by E‐Prime 3 software (Psychology Software Tools; Pittsburgh, PA). After signing informed consent, participants were seated in a comfortable chair and equipped with the physiological sensors. Participants first rated two positive (happy, joyful) and two negative items (sad, downcast) of the UWIST scale (Matthews et al., 1990) to assess their momentary mood before the manipulations (“Right now, I'm feeling…”). Ratings were made on scales ranging from 0 (*not at all*) to 100 (*very much*) using a slider. To prevent suspicion, the affect measure was introduced as a standard assessment because people enter the laboratory in different states. Later we created mood scores by summing the positive affect ratings and the reverse‐coded ratings of the negative items, resulting in scores ranging from 0 (*extremely negative mood*) to 400 (*extremely positive mood*). We assessed affect to mirror a possible link between mood states and cardiovascular activity (see Gendolla, Brinkmann, et al., 2012; Gendolla & Brinkmann, 2005, for reviews).

Next, participants were introduced to the general protocol, which began with 24 trials of a Stroop (1935) color‐naming task with vocal responses. To assess response accuracy, the experimenter remained in the experimental room during this part of the study and noted participants' responses. Reaction times were assessed using a Serial Response Box (SRBox, Psychology Software Tools, Inc) equipped with a microphone (ATR20, Audio‐Technica, Inc). The Stroop task comprised 12 congruent and 12 incongruent trials that occurred in a fixed, previously determined random order. The color words appeared in red, blue, yellow, and green. Trials began with a fixation cross (1000 ms), followed by either a congruent or an incongruent Stroop item that remained on the screen until a response was given (max 3000 ms), followed by the feedback “answer recorded” or “please answer faster” (1500 ms).

**FIGURE 1:**
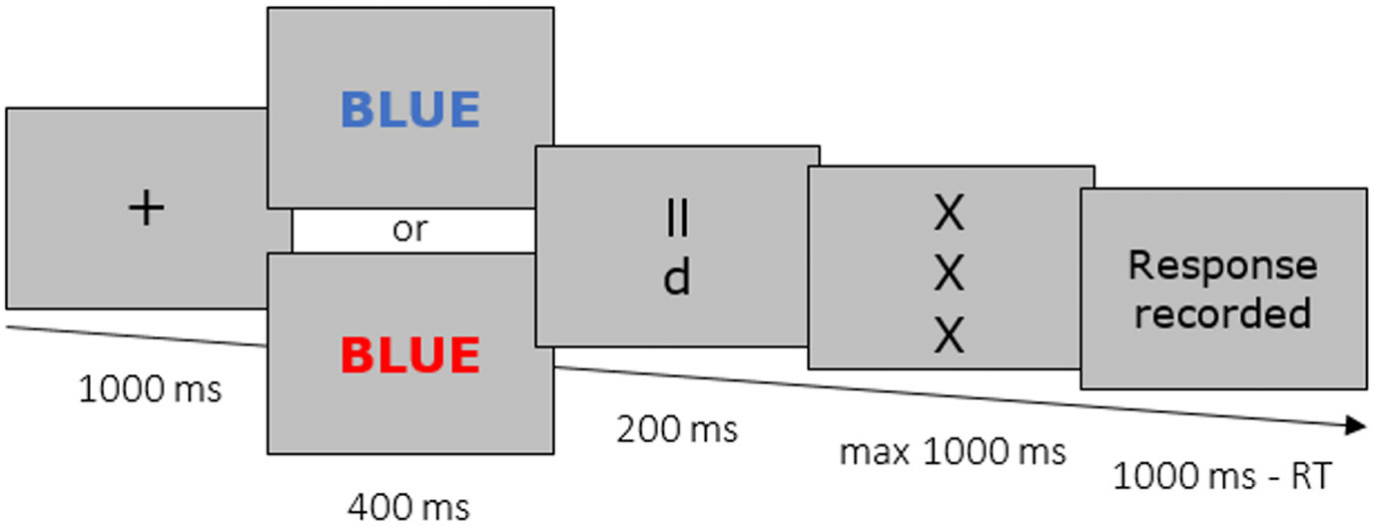
Example of a task trial in Experiment 1. The type of Stroop prime (congruent or incongruent) depends on the experimental condition.

After completion of the Stroop task, the experimenter went to an adjacent control room and participants watched an 8‐min hedonically neutral documentary video about Swiss mountains to assess their cardiovascular baseline activity. Next, we administered a 5‐min computerized version of a relatively easy d2 attention task (Brinkenkamp, 1981) with Stroop primes. Participants were instructed to respond correctly and as fast as possible. As depicted in Figure 1, trials began with a fixation cross (1000 ms) followed by either a congruent (i.e., non‐conflict‐related) or an incongruent (i.e., conflict‐related) Stroop prime (depending on individuals' experimental condition) that appeared for 400 ms. Next, participants saw a d2 task stimulus (200 ms) followed by a mask that remained on the screen until participants answered within 1000 ms. Participants indicated whether the stimulus was a “d” with exactly two apostrophes, which were displayed either above and/or below the target letter, by pressing “yes” and “no” response keys with two fingers of their dominant hand. Incorrect trials presented the letters “d” or a “p” with 0, 1, or 3 apostrophes. If a response was entered within 1000 ms, the message “response recorded” appeared on the screen, otherwise “please answer faster” was displayed. To avoid potential feedback‐related affective reactions (e.g., Kreibig et al., 2012) that could interfere with the primed conflict manipulation, we did not give correctness feedback during the main task. The inter‐trial interval randomly varied between 500 and 1000 ms. The task comprised 84 trials and took 5 min.

Before the d2 task, participants completed 12 practice trials with correctness feedback. Practice trials started with the presentation of colored neutral words (the French words for tree, truck, chair, bicycle, and table) instead of Stroop primes, to minimize participants' habituation to the later administered primes. Prior to the task, a message announced that in the following main task words of the preliminary task would be displayed (without mentioning their congruent or incongruent nature).

After the task, participants rated their momentary affective state again, answered biographical questions (gender, age, etc.) and questions about possible medication and their cardiovascular health status. Finally, participants were thanked and debriefed.

### 2.2 Results and discussion

#### 2.2.1 Cardiovascular baselines

Complying with Shapiro et al.'s (1996) recommendation to average at least 3 blood pressure measures, and because cardiovascular activity decreases toward the end of habituation periods, we had a priori decided to create baseline scores of PEP, HR, SBP, and DBP by averaging values assessed during the last 3 min of the habituation period (Cronbach's *α*s > .96). Means and standard errors appear in Table 1. Preliminary analyses revealed that the difference in SBP baseline values between the later Stroop Prime conditions approached significance, *t*(82) = 1.91, *p* = .06, *η* ^2^ = .04. We thus tested below if the SBP baselines were significantly related to SBP reactivity. There were no other significant baseline differences between the later conditions (*t*s <1.05, *p*s > .295).^2^

**TABLE 1:** Means and standard errors (in parentheses) of the cardiovascular baseline scores (Study 1)

	Incongruent Stroop primes	Congruent Stroop primes
PEP	99.35 (1.33)	101.66 (1.78)
SBP	110.16 (1.46)	106.13 (1.53)
DBP	60.20 (0.60)	60.20 (1.10)
HR	76.45 (1.66)	74.70 (1.91)

Abbreviations: DBP, diastolic blood pressure (in mmHg); HR, heart rate (in bpm); PEP, pre‐ejection period (in ms); SBP, systolic blood pressure (in mmHg).

#### 2.2.2 Cardiovascular reactivity

Following Llabre et al. (1991), we calculated reactivity scores for each cardiovascular parameter by subtracting individuals' baseline values from the five 1‐min cardiovascular activity scores assessed during task performance. Then, we averaged the reactivity scores across the 5 min (Cronbach's *α*s > .89). Next, we conducted preliminary ANCOVAs of these reactivity scores to test for potential associations with the respective baseline values. Results showed a significant positive association between the HR baseline and reactivity scores, *F*(1,78) = 6.80, *p* = .011, *η* ^2^ = .08, (regression slopes did not significantly differ between the conditions, *p* = .759). We therefore included the HR baseline scores as covariate in the HR reactivity analysis. No other associations between baseline and reactivity scores were significant (*p*s > .40).

##### PEP reactivity

**FIGURE 2:**
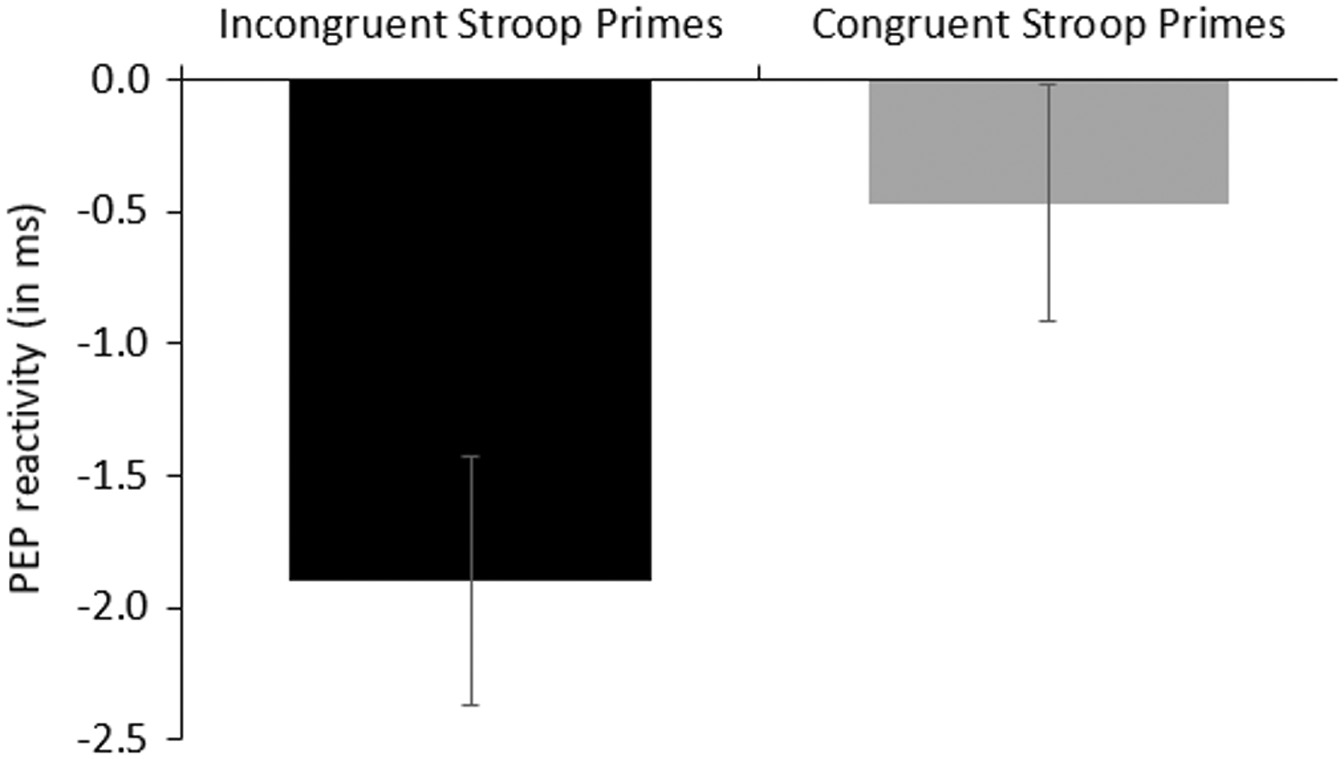
PEP (in ms) reactivity as a function of the Stroop prime condition (Experiment 1). Shorter PEP reflects higher effort.

We tested our main hypothesis with an independent samples *t* test with the Stroop Prime condition as independent variable and PEP reactivity as dependent measure. The result supported our prediction, *t*(78) = 2.20, *p* = .031, *η* ^2^ = .06. As expected and depicted in Figure 2, participants in the incongruent, conflict‐related Stroop Prime condition (*M* = −1.90, *SE* = 0.47) showed significantly stronger PEP reactivity—meaning higher effort—than those who were exposed to congruent, non‐conflict‐related Stroop primes (*M* = −0.47, *SE* = 0.45).

##### SBP, DBP, and HR reactivity

Means and standard errors appear in Table 2. No significant effects were found on DBP responses (*p* = .428) or baseline‐adjusted SBP (*p* = .122) and HR reactivity (*p* = .520).

**TABLE 2:** Means and standard errors (in parentheses) of blood pressure and heart rate reactivity scores (Study 1)

	Incongruent Stroop primes	Congruent Stroop primes
SBP^a^	2.03 (0.62)	3.43 (0.64)
DBP	1.97 (0.62)	2.64 (0.57)
HR^a^	1.54 (0.49)	2.00 (0.50)

Abbreviations: DBP, diastolic blood pressure (in mmHg); HR, heart rate (in bpm); SBP, systolic blood pressure (in mmHg).

^a^ Baseline adjusted.

#### 2.2.3 Initial Stroop task performance

We conducted within persons *t* tests (Stroop trials: congruent vs. incongruent) of participants' performance on the initial Stroop task, which revealed a strong Stroop effect: responses were more accurate (*M* = 99%, *SE* = 0.28 vs. *M* = 93%, *SE* = 1.19)—*t*(83) = 5.49, *p* < .001, $\eta_{p}^{2}$ = .27—and faster (*M* = 682 ms, *SE* = 18 vs. *M* = 823 ms, *SE* = 22)—*t*(83) = 8.48, *p* < .001, $\eta_{p}^{2}$ = .46—in congruent than in incongruent Stroop trials. This shows that the initial Stroop task worked as expected and that participants experienced the difference between incongruent, conflict‐related versus congruent, non‐conflict‐related Stroop trials.

#### 2.2.4 Task performance, affect, and task difficulty ratings

Regarding the d2 attention task, participants were highly accurate (average *M* = 88%, *SE* = 1.29) and fast (average *M* = 394 ms, *SE* = 11), meaning that the task was as intended relatively easy. Independent samples *t* tests of task performance and the self‐report measure of task difficulty (average *M* = 37, *SE* = 3) revealed no significant differences between the conflict‐related and non‐conflict‐related Stroop Primes conditions (*t*s <1.43, *p*s > .157).

To assess participants' affective states, we calculated mood sum scores based on the measure taken before (average *M* = 296, *SE* = 7) and after (average *M* = 290, *SE* = 7) the task (negative items were reverse coded; Cronbach's *α*s > .79). A 2 (Stroop Prime) × 2 (Time) mixed model ANOVA of participants' affect scores did not reveal any significant effects (*F*s <2.82, *p*s > .097).

#### 2.2.5 Conclusions

We found the predicted primed cognitive conflict effect on PEP reactivity—our most sensitive effort measure—during the performance of a cognitive task that was neither difficult nor conflict‐related itself. That is, we found evidence that conflict itself is indeed effortful. Importantly, with our experimental procedure we could disentangle the cognitive conflict effect on effort from a mere objective response difficulty effect on resource mobilization. We ran Study 2 to conceptually replicate and extend this new finding.

## 3 EXPERIMENT 2

Our second experiment served two purposes. First, we administered a relatively easy short‐term memory task adapted from Bijleveld (2018) to conceptually replicate and generalize the primed cognitive conflict effect on PEP found in Experiment 1. Second, we tested whether giving participants the opportunity to personally choose task characteristics could eliminate the primed conflict effect on PEP reactivity. Choice can have many beneficial effects (see Leotti et al., 2010; Patall et al., 2008, for reviews). Most relevant, following the logic of an action‐shielding model (Gendolla et al., 2021), personal task choice can shield effort against incidental affective influences like happy or sad music (Falk et al., 2022a, 2022b). This action‐shielding effect can be attributed to an implemental mindset (Gollwitzer, 1990), which is activated by intention formation (Gollwitzer et al., 1990; Heckhausen & Gollwitzer, 1987)—choosing actions or aspects of action execution—and enhances commitment (Bouzidi et al., 2022; Nenkov & Gollwitzer, 2012) and task‐focus (Kuhl, 1986). We expected that the personal choice of task characteristics should shield against the aversiveness of cognitive conflict in the same way as it can shield against other incidental affective stimulations (Falk et al., 2022a, 2022b; Gendolla et al., 2021). Finding the shielding effect of personal choice would further speak for the idea that cognitive conflict influences effort because of its aversiveness and the corresponding effect on subjective demand.

To test our shielding hypothesis, we extended our experimental design. Participants in a Self‐Chosen Characteristics condition could decide between four different typefaces in which the stimuli of the administered short‐term memory task would be presented. By contrast, following a yoked design, the typeface was assigned for participants in the Assigned Characteristics condition. Corresponding to Experiment 1, we expected that primed cognitive conflict should increase cardiac response in the Assigned Characteristics condition. By contrast, participants in the Self‐Chosen Characteristics condition should be shielded against the cognitive conflict aversiveness effect on effort, resulting in weak PEP responses in both the Congruent and Incongruent Prime conditions. This should happen because a relatively easy task only necessitates low effort. Consequently, according to the principles of motivational intensity theory (Brehm & Self, 1989), cardiac reactivity in the Chosen Characteristics condition was predicted to be low, even though choice increases commitment (e.g., Bouzidi et al., 2022).^3^ Overall, this resulted in the prediction of a 3:1 pattern with a stronger cardiac PEP response in the Assigned‐Characteristics/Incongruent condition than in the other three conditions.

### 3.1 Method

The apparatus, physiological measurement, and basic procedure were identical with our first study; only some details of the priming procedure differed. We also eliminated obsolete measures to prevent any suspicion regarding the study aims. Consequently, we did not assess participants' mood as this measure had not revealed any effects in Experiment 1.

#### 3.1.1 Participants and design

Based on the results of Experiment 1, we aimed to collect valid data of at least 30 participants per condition in the present 2 (Stroop Prime: incongruent vs. congruent) × 2 (Choice: self‐chosen vs. assigned) between‐persons design. To compensate for possible data loss, we recruited 135 first year psychology students from the University of Geneva. *N* = 131 of the invited participants (average age 22 years) showed up in the experiment and received partial course credit. Participants were randomly assigned to one of the 4 conditions. The gender distributions were balanced (Self‐Chosen/Incongruent‐Prime: 27 women, 6 men; Self‐Chosen/Congruent‐Prime: 28 women, 5 men, 1 other; Assigned/Incongruent‐Prime: 27 women, 6 men; Assigned/Congruent‐Prime: 26 women, 5 men). We excluded one participant from the analyses because his PEP reactivity was more than 3 *SD*s higher than the condition mean, leaving a sample of *N* = 130. Technical problems prevented us from assessing ICG signals of 6 participants and blood pressure of 1 participant. One other participant was excluded because of his poor performance (response accuracy <50%), suggesting misunderstood instructions. A G*Power (Faul et al., 2007) sensitivity analysis revealed that our sample size was big enough to detect significant effects of medium size with 80% power in our 2 × 2 between‐persons design.

Therefore, the final samples for PEP/HR and SBP/DBP analyses were *N* = 123 and *N* = 128, respectively. There were again no significant differences between the conditions regarding age, gender, or body mass index (*p*s > .130, see Table S4 in the Supporting Information).

#### 3.1.2 Procedure

The initial vocal Stroop task and cardiovascular baseline activity assessment were identical with Experiment 1. In addition, participants in the Self‐Chosen Characteristics condition read a cover story to give their choice a meaning (see Patall et al., 2008). Specifically, they read “research has shown that individuals perform better on the task when stimuli are presented in the typeface of their choice”. Therefore, they could choose between four different typefaces (Rockwell, OCR A Extended, Copperplate Gothic Bold, or Gadugi) presented for 1‐min on the next screen to give them time to deliberate. At the end of this period, participants indicated their decision by pressing a font‐corresponding key. Once their choice was made, they were asked whether they were sure about their choice to assure their choice‐related commitment. By pressing “yes”, the procedure continued; by pressing “no”, they had to choose and confirm their decision again. In the Assigned Characteristics condition, participants were yoked with a participant in the Self‐Chosen Characteristics condition. For instance, if a participant had chosen the font ‘Gadugi’, the next participant in the Assigned condition read “research has shown that individuals perform better on the task when stimuli are presented in this font”. That is, the typeface assignment had ostensibly the same positive effects on performance as font choice in the Self‐Chosen Characteristics condition. Only the freedom of choice differed. To further match the conditions as much as possible, Assigned Characteristics participants had a 1‐min break with the assigned typeface displayed on the screen. Then, to assess the subjective feeling of choice, all participants answered the question “To what extend could you decide the characteristics of the task?” on a continuous scale with a slider (ranging from 0 – *not at all* to 100 – *very much*).

Next, participants performed a relatively easy short‐term memory task adapted from Bijleveld (2018) with embedded congruent or incongruent Stroop primes. Trials began with a fixation cross (1000 ms), followed by a first series of 5 random digits (e.g., “43,861”) presented for 500 ms. Next, either a congruent or an incongruent Stroop prime appeared for 1000 ms and was directly followed by a second series of 5 digits (2000 ms), which was either identical with the first series or not (50% ratio). In unidentical trials, only one digit differed between the two series. Participants determined whether the first and the second series were identical or not by pressing a “yes” or “no” response key with two fingers of their dominant hand. “Response recorded” was displayed following a response; “Please respond faster” appeared on the screen if participants did not answer within a 2000 ms response time window. The inter‐trial interval randomly varied between 500 and 1500 ms. The task comprised 48 trails and took 5 minutes. Before the main task, participants completed 6 practice trials with correctness feedback; no correctness feedback was given in the main task. As in Experiment 1, task difficulty was assessed after the main task. The procedure finished again with biographical questions and a debriefing.

### 3.2 Results and discussion

#### 3.2.1 Cardiovascular baselines

Cardiovascular baseline scores of PEP, HR, SBP, and DBP were again created by averaging values assessed during the last 3 min of the habituation period (Cronbach's *α*s > .95). Means and standard errors appear in Table 3.^4^

**TABLE 3:** Means and standard errors (in parentheses) of the cardiovascular baseline scores (Study 2)

	Assigned characteristics		Self‐chosen characteristics	
	Incongruent Stroop primes	Congruent Stroop primes	Incongruent Stroop primes	Congruent Stroop primes
PEP	98.75 (1.81)	104.41 (2.55)	97.72 (1.64)	102.64 (1.98)
SBP	106.54 (1.61)	105.42 (1.49)	106.20 (1.83)	107.16 (1.86)
DBP	60.67 (0.96)	58.68 (0.92)	59.32 (0.87)	60.05 (0.93)
HR	83.97 (1.68)	78.81 (2.32)	84.56 (2.09)	80.43 (2.66)

Abbreviations: DBP, diastolic blood pressure (in mmHg); HR, heart rate (in bpm); PEP, pre‐ejection period (in ms); SBP, systolic blood pressure (in mmHg).

#### 3.2.2 Cardiovascular reactivity

As in Experiment 1, we subtracted participants' baseline values from the five 1‐min cardiovascular activity scores assessed during task performance to create reactivity scores for each cardiovascular index (Cronbach's *α*s > .92). Next, we conducted preliminary 2 (Stroop Prime) × 2 (Choice) ANCOVAs of these reactivity scores to test for potential associations with the respective baseline scores. No such association was significant (*F*s <2.39, *p*s > .124).

##### PEP reactivity

**FIGURE 3:**
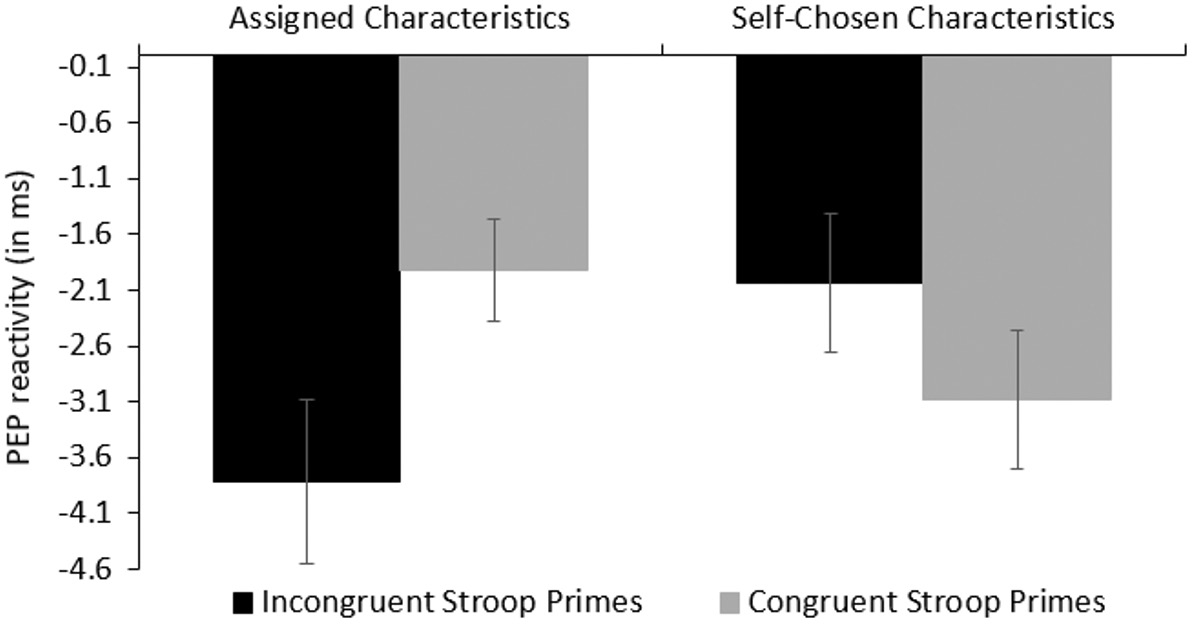
PEP reactivity (in ms) as a function of the Stroop Prime and Choice conditions (Experiment 2). Shorter PEP reflects higher effort.

We tested our theory‐based main hypothesis with an a priori contrast analysis—the most powerful and thus most appropriate statistical tool to test specific interaction patterns of cell means (Rosenthal & Rosnow, 1985; Wilkinson & The Task Force on Statistical Inference of APA, 1999). We expected a 3:1 pattern with stronger reactivity in the Assigned/Congruent Prime condition (weight −3) than in the other three conditions (weights +1). In support of our hypothesis, the contrast was significant—*F*(1,119) = 4.27, *p* = .041, *η* ^2^ = .04—and the pattern of cell means occurred as expected, as depicted in Figure 3.

We also conducted additional focused cell contrasts, which revealed that PEP reactivity in the Assigned/Incongruent Prime condition (*M* = −3.81, *SE* = 0.73) was significantly stronger than the Assigned/Congruent Prime condition (*M* = −1.92, *SE* = 0.45), *t*(119) = 2.11, *p* = .018 (one‐tailed),^5^ *η* ^2^ = .04. This replicated the primed cognitive conflict effect on PEP reactivity we had found in Experiment 1 when the task characteristics were assigned. PEP reactivity in the Assigned/Incongruent Prime condition was also significantly stronger than in the Self‐Chosen/Incongruent Prime condition (*M* = −2.04, *SE* = 0.62), *t*(119) = 2.07, *p* = .020, *η* ^2^ = .03. The difference between the Assigned/Incongruent‐Prime and the Self‐Chosen/Congruent‐Prime condition (*M* = −3.09, *SE* = 0.63) was not significant (*t* = 0.82, *p* = .207), although the pattern followed our prediction. Furthermore, cell comparisons between the Assigned/Congruent Prime, Self‐Chosen/Incongruent Prime, and Self‐Chosen/Congruent Prime conditions revealed no significant differences (*t*s <1.26, *p*s > .21). That is, primed conflict had no significant effect on PEP reactivity when task characteristics could be chosen.

##### SBP, DBP, and HR reactivity

Means and standard errors appear in Table 4. The a priori contrasts for HR and blood pressure responses were not significant (*F*s <0.78, *p*s > .378).

**TABLE 4:** Means and standard errors (in parentheses) of systolic blood pressure, diastolic blood pressure, and heart rate reactivity during task performance as function of Stroop primes and Choice condition (Study 2)

	Assigned characteristics		Self‐chosen characteristics	
	Incongruent Stroop primes	Congruent Stroop primes	Incongruent Stroop primes	Congruent Stroop primes
SBP	4.39 (1.08)	2.75 (0.95)	4.64 (0.72)	3.38 (0.66)
DBP	2.39 (0.77)	3.22 (0.46)	2.77 (0.59)	3.09 (0.76)
HR	2.16 (0.95)	1.75 (0.94)	2.27 (0.88)	2.56 (1.06)

Abbreviations: DBP, diastolic blood pressure (in mmHg); HR, heart rate (in bpm); SBP, systolic blood pressure (in mmHg).

#### 3.2.3 Initial Stroop task performance

Within‐persons *t* tests (Stroop trials: congruent vs. incongruent) of participants' performance in the initial Stroop task replicated the Stroop effect found in Experiment 1: responses were more accurate (*M* > 99%, *SE* <0.01 vs. *M* = 97%, *SE* = 0.01), *t*(127) = 4.71, *p* < .001, $\eta_{p}^{2}$ = .15, and faster (*M* = 1139 ms, *SE* = 34 vs. *M* = 1194 ms, *SE* = 34)—*t*(127) = 2.05, *p* = .042, $\eta_{p}^{2}$ = .03—in congruent than in incongruent Stroop trials.

#### 3.2.4 Memory task performance

Participants made by average *M* = 82% (*SE* = 0.78) correct responses in the short‐term memory task (reaction time: average *M* = 857, *SE* = 15), suggesting that the main task was as intended relatively easy. The 2 (Stroop Prime) × 2 (Choice) ANOVAs did not reveal any significant effects on task performance (*F*s <1.52, *p*s > .210).

#### 3.2.5 Task difficulty ratings and choice manipulation check

A 2 (Stroop Prime) × 2 (Choice) ANOVA of the choice manipulation check revealed a strong significant Choice main effect, *F*(1,124) = 43.59, *p* < .001, *η* ^2^ = .26. As intended, participants in the Self‐Chosen Characteristics condition (*M* = 52.03, *SE* = 2.78) experienced much more freedom in choosing task characteristics than participants in the Assigned Characteristics condition (*M* = 25.16, *SE* = 2.93). Other effects were not significant (*F*s <0.36, *p*s > .551). No significant effects emerged on participants' ratings of subjective task difficulty, *F*s <0.69, *p*s > .406, (average *M* = 53.31, *SE* = 2.06).

#### 3.2.6 Conclusions

This study found evidence for the moderating effect of personal choice on primed cognitive conflict's effect on effort assessed as PEP response. First, replicating the conflict effect found in Experiment 1, participants who were in the Assigned Characteristics condition showed stronger PEP responses when they faced conflict‐related primes than when they processed non‐conflict‐related primes in a task that was neither difficult nor conflict‐related itself. That is, we could again disentangle the conflict effect from mere response difficulty effects and thus show once more that cognitive conflict itself was indeed effortful. Second, giving participants the opportunity to personally choose task characteristics eliminated this effect. That is, the personal choice of task characteristics could as expected shield resource mobilization against the influence of cognitive conflict.

## 4 GENERAL DISCUSSION

Many researchers have posited that cognitive conflict is effortful (e.g., Dignath et al., 2020; Dreisbach & Fischer, 2012a; Inzlicht et al., 2015; Kool et al., 2013; Kool & Botvinick, 2018; van Steenbergen, 2015). However, evidence for the effortfulness of cognitive conflict itself was not conclusive to date. We think this was the case because of the usually applied strategy to investigate cognitive conflict effects on effort with cognitive control tasks that had originally been designed to assess performance effects rather than objective physiological indices of resource mobilization. In such tasks, responses in conflict‐related incongruent trials are objectively more difficult than congruent trials, meaning a confound between cognitive conflict and mere objective response difficulty. We conducted the two present experiments to resolve this problem by applying an experimental strategy developed by Dreisbach and Fischer (2012a). Instead of inducing manifest response conflict we primed cognitive conflict. Both present studies found that cognitive conflict itself is indeed effortful.

Our Experiment 1 revealed, as predicted, a significant conflict prime effect on participants' PEP responses during the performance on an attention task that was itself neither difficult nor conflict‐related. The activation of participants' mental representations of cognitive conflict with pictures of incongruent Stroop task trials resulted in stronger PEP responses than exposure to non‐conflict‐related primes. Experiment 2 conceptually replicated this primed cognitive conflict effect on cardiac response when the characteristics of a short‐term memory task were assigned—as they were in Experiment 1 and other research on cognitive control. As expected, letting participants personally choose task characteristics eliminated the conflict prime effect on PEP reactivity. This shielding effect conceptually replicated the results of recent studies on an action‐shielding model, in which personal choice immunized against the effect of affect‐inducing music on PEP responses (Falk et al., 2022a, 2022b; Gendolla et al., 2021).

We interpret the present conflict prime and shielding effects as further support for the idea that cognitive conflict is effortful because of its affective component—conflict is aversive (Berger et al., 2020; Dignath et al., 2020; Dreisbach & Fischer, 2012a, 2012b; Inzlicht et al., 2015; Pourtois et al., 2020; Silvestrini & Gendolla, 2019; van Steenbergen et al., 2010). Aversiveness can influence effort. There is ample evidence that both experienced and implicit negative affect—especially sadness and fear—increases subjective task demand and thus effort‐related physiological responses in cognitive tasks—as long as success is possible and justified (see Gendolla, 2012; Gendolla & Brinkmann, 2005; Gendolla, Brinkmann, et al., 2012, for reviews). However, as already shown for other affective influences on action execution, personal choice of task characteristics could minimize the effect of cognitive conflict on effort. This shielding effect can be attributed to an implemental mindset (Gollwitzer, 1990), which is activated by intention formation (Gollwitzer et al., 1990; Heckhausen & Gollwitzer, 1987), and enhances commitment (Bouzidi et al., 2022; Nenkov & Gollwitzer, 2012) and task‐focus (Kuhl, 1986). Most importantly, it further speaks for the idea that cognitive conflict is effortful because it is aversive and thus influences subjective demand.

On the physiological level, we found significant conflict effects on PEP reactivity, but not on blood pressure or HR. This is not surprising, as PEP is the most sensitive noninvasive measure of β‐adrenergic sympathetic impact and thus effort (see Kelsey, 2012; Richter et al., 2008; Wright, 1996). Blood pressure and HR are less directly influenced by β‐adrenergic sympathetic impact and thus noisier effort measures. Importantly, we did not find significant decreases in HR or DBP during task performance. Accordingly, PEP reactivity can have hardly been affected by cardiac preload or vascular afterload effects instead of β‐adrenergic sympathetic activity (Sherwood et al., 1990). That is, the present PEP effects can be interpreted as reflecting effort.

On the behavioral level, we have not found significant effects on response accuracy or response speed during participants' performance on the administered attention and short‐term memory tasks. Accordingly, there was no evidence for a link between effort and performance. However, it is of note that effort intensity (behavioral input) and performance (behavioral output) are not conceptually identical and performance depends besides effort also, or even more, on task‐related capacity and strategies (Locke & Latham, 1990). Consequently, one cannot expect that variations in effort intensity always result in performance effects. Nevertheless, participants' overall high response accuracy supports our assumption that we successfully created relatively easy tasks, as further supported by our verbal task difficulty measures. Considering this, the lack of performance effects is still less surprising, because effort may be necessary to perform well in demanding tasks, but it is not necessary for succeeding in easy and moderately difficult tasks (e.g., Gendolla & Krüsken, 2001). In contrast to the usually administered rather difficult cognitive control tasks in cognitive conflict research, we sought to disentangle conflict effects from mere objective response difficulty effects on effort. We did so by administering cognitive tasks that were neither difficult nor conflict‐related themselves. Consequently, our present conflict priming effects on PEP reactivity cannot be attributed to objective response difficulty. Rather, conflict itself influenced effort.

Regarding the role of experienced affect, Experiment 1 did not reveal a conflict effect on participants' mood states assessed after the task with the conflict primes. Also this is not surprising. Experienced affective states can influence cognitive conflict adaptation (e.g., Berger et al., 2019; van Steenbergen et al., 2009, 2010, 2012), but there seems to be no evidence that cognitive conflict has effects on consciously experienced mood states (see Dignath et al., 2020, for a review). Rather, affective reactions to low‐order cognitive conflict occur fast, are short, and apparently implicit. Directly referring to our primed conflict manipulation, pictures of incongruent Stroop task trials as conflict primes have been shown to activate the concept of negativity in behavioral sequential priming studies (e.g., Dreisbach & Fischer, 2012a) and to result in implicit negative evaluations in the affective‐misattribution task (e.g., Damen et al., 2018). But the conflict primes did not elicit conscious negative feelings in those studies. Apparently, the conflict primes are implicitly aversive. This suggests that the conflict primes influenced effort in the same way as other types of negative affect primes by influencing subjective task demand rather than objective response difficulty. Research in the context of the Implicit‐Affect‐Primes‐Effort (IAPE) model (Gendolla, 2012, 2015) has provided replicated evidence that priming sadness or fear during easy‐to‐moderately difficult tasks leads to stronger cardiac responses than priming happiness (e.g., Chatelain & Gendolla, 2015; Gendolla & Silvestrini, 2011; Lasauskaite et al., 2013; Silvestrini & Gendolla, 2011). This happens via the implicit activation of ease (in the case of happiness or anger) and difficulty (in the case of sadness or fear) concepts (Lasauskaite et al., 2017) without effects on conscious feelings. We assume that conflict primes influence effort in the same way, meaning that conflict is effortful because it is aversive, mentally associated with difficulty, and thus increases subjective demand online during task performance.

Finally, one could argue that there were far more women than men in our studies, which could have biased our results. However, it is of note that gender differences in cognitive conflict were not of interest in our studies and we are not aware of any evidence that would have led us to take gender as a moderator variable. Most relevant, the gender distributions were balanced across conditions in both studies. Therefore, even if participants' gender could have biased our results, this could not explain the here reported effects as there was no confound between gender and the experimental conditions.

### 4.1 Outlook and conclusions

The results of our two present experiments show for the first time that cognitive conflict itself rather than objective response difficulty influences cardiac response. That way, our studies further advocate for the cognitive conflict‐triggered affective signal hypothesis (see Dignath et al., 2020). Although these findings directly concern the effortfulness of lower‐order cognitive conflict, they also have implications for higher‐order conflict. One can wonder whether conflicts that are not inherent in given tasks can also influence ongoing behavior and resource mobilization in everyday life. This calls for studies on the impact of higher‐order conflict on effort—for example in the context of individual's long term versus momentary hedonic goals (e.g., Bernecker & Becker, 2021), motivational study‐leisure conflicts (see Brassler et al., 2016; Duckworth et al., 2019; Fries & Dietz, 2007), or action crises (see Brandstätter & Schüler, 2013; Herrmann & Brandstätter, 2015). From this perspective, our present two studies may be the starting point for research addressing if and how cognitive conflict can influence resource mobilization in everyday life.

## AUTHOR CONTRIBUTIONS

**Yann S. Bouzidi:** Conceptualization; data curation; formal analysis; investigation; methodology; software; writing – original draft. **Guido H. E. Gendolla:** Funding acquisition; investigation; methodology; supervision; validation; writing – review and editing.

## Supporting information


**Table S1** Means and standard errors (in parentheses) of demographic data (Experiment 1)
**Table S2** Means and standard errors (in parentheses) of baseline scores (Experiment 1)
**Table S3** Means and standard errors (in parentheses) of cardiac output and total peripheral resistance reactivity (Experiment 1)
**Table S4** Means and standard errors (in parentheses) of demographic data (Experiment 2)
**Table S5** Means and standard errors (in parentheses) of baseline scores (Experiment 2)
**Table S6** Means and standard errors (in parentheses) of cardiac output and total peripheral resistance reactivity (Experiment 2)

## Data Availability

The data and data coding for the here reported studies are available on Yareta—the open access data archiving server of the University of Geneva: https://doi.org/10.26037/yareta:zvnvr2ajz5fvrlaqoge5pqvl3e. Readers interested in the raw data are welcome to contact us directly.

## Footnotes

^1^
It is of note that anger—another type of specific negative affect—is linked to high coping potential (e.g., Lerner & Keltner, 2001). Consequently, anger decreases subjective task difficulty and effort in easy to moderately difficult tasks (e.g., Chatelain & Gendolla, 2015; Gendolla & Silvestrini, 2011).

^2^
For readers interested in gender differences in cardiovascular activity, we compared the baseline values of women and men with *t* tests (including gender in two‐factorial ANOVAs was not an option, because there were far more women than men in our sample). There was a significant gender difference in the SBP baseline values, *t*(82) = 5.29, *p* < .001, *η* ^2^ = .25, reflecting higher values for men (*M* = 115.77, *SE* = 1.98) than for women (*M* = 104.98, *SE* = 1.03), which is usual (e.g., Martins et al., 2001). No further gender differences in cardiovascular baseline scores were significant (*t*s <1.03, *p*s > .306). The only gender difference in cardiovascular reactivity emerged for DBP, *t*(82) = 2.67, *p* = .009, *η* ^2^ = .08, due to stronger responses in men (*M* = 3.97, *SE* = 0.77) than in women (*M* = 1.59, *SE* = 0.48) (other *t*s <0.98, *p*s > .332).

^3^
Note that effort in the Self‐Chosen Characteristics condition should only be high when a task is objectively difficult or if difficulty is unclear, because high and unclear difficulty leads to high effort when the latter is justified. By contrast, easy‐to‐moderately‐difficult tasks only necessitate low effort—if affective influences are neutralized.

^4^
For interested readers, we again compared the baseline values of women and men with *t* tests—including gender as additional factor in ANOVAs was no option as our sample comprised far more women than men. We found again only a gender difference in SBP baselines, *t*(126) = 5.20, *p* < .001, *η* ^2^ = .18, reflecting higher values for men (*M* = 114.93, *SE* = 2.20) than for women (*M* = 104.46, *SE* = 0.81), (other *t*s <0.87, *p*s > .386). There were also significant gender differences in responses of PEP, *t*(121) = 2.53, *p* = .013, *η* ^2^ = .05, (men *M* = −4.42, *SE* = 1.05; women *M* = −2.37, *SE* = 0.31), SBP, *t*(126) = 3.11, *p* = .002, *η* ^2^ = .07, (men *M* = 6.62, *SE* = 1.09; women *M* = 3.20, *SE* = 0.46), and HR, *t*(120) = 2.79, *p* = .006, *η* ^2^ = .06, (men *M* = 4.94, *SE* = 1.51; women *M* = 1.59, *SE* = 0.46).

^5^
The *p*‐values of the focused cell contrasts testing directed predictions are one‐tailed.
