# Supplementary material for: Is cognitive conflict really effortful? Conflict priming and shielding effects on cardiac response
Source: Psychophysiology. 2022 Sep 8;60(2):e14169. doi: 10.1111/psyp.14169 (PMC10078432; doi:10.1111/psyp.14169)
Supplement: Supplementary file 1 — Table S1 Means and standard errors (in parentheses) of demographic data (Experiment 1) Table S2 Means and standard errors (in parentheses) of baseline scores (Experiment 1) Table S3 Means and standard errors (in parentheses) of cardiac output and total peripheral resistance reactivity (Experiment 1) Table S4 Means and standard errors (in parentheses) of demographic data (Experiment 2) Table S5 Means and standard errors (in parentheses) of baseline scores (Experiment 2) Table S6 Means and standard errors (in parentheses) of cardiac output and total peripheral resistance reactivity (Experiment 2) [file PSYP-60-0-s001.docx]

**Online Supplementary Material**

**Is Cognitive Conflict Really Effortful? Conflict Priming and
Shielding Effects on Cardiac Response**

Yann S. Bouzidi and Guido H.E. Gendolla

University of Geneva, Switzerland

For researchers who are interested in a fuller picture of hemodynamic responses, we also assessed cardiac output (CO) and total peripheral resistance (TPR). Nonetheless, it is of note that these two indices were not relevant for our hypotheses. CO was assessed with the ICG monitor and calculated by the Cardioscreen system according to the Sramek and Bernstein formula (Bernstein, 1986). TPR was calculated from CO and mean arterial pressure (MAP = [2 x DBP + SBP] / 3) by using the formula TPR = (MAP / CO) * 80 (Sherwood et al., 1990).

**1. Experiment 1**

| Supplementary Table 1  *Means and Standard Errors (in Parentheses) of Demographic data (Experiment 1).* | | |
| --- | --- | --- |
|  | *Incongruent Stroop Primes* | *Congruent Stroop Primes* |
| Gender | 31 women / 15 men | 30 women / 14 men |
| Age | 24.91 (0.85) | 23.84 (0.74) |
| BMI | 22.09 (0.58) | 21.89 (0.48) |
| Note. BMI = body mass index; Med car = drugs that influence cardiovascular system; Med dep = antidepressant drugs | | |

**1.1 CO and TPR Baseline Values** We constituted CO and TPR baseline scores by averaging cardiovascular values of the last 3 minutes of the habituation period, which showed high internal consistency during the last three minutes (Cronbach’s αs > .99). Means and standard errors appear in Supplementary Table 1.

| Supplementary Table 2  *Means and Standard Errors (in Parentheses) of Baseline Scores (Experiment 1).* | | |
| --- | --- | --- |
|  | *Incongruent Stroop Primes* | *Congruent Stroop Primes* |
| CO | 5.67 (0.17) | 5.17 (0.19) |
| TPR | 1120.24 (31.18) | 1220.97 (44.72) |
| Notes. CO = cardiac output (in liters per minute), TPR = total peripheral resistance (in dynes second per centimeter to the 5^th^ power). | | |

Preliminary analyses revealed marginal a priori condition differences for the baselines of CO, *t*(78) = 1.98, *p* = .051, η^2^ = .05, and TPR, *t*(78) = 1.88, *p* = .064, η^2^ = .04. We thus considered these baseline scores as covariates in the reactivity score analyses below. Moreover, there was a gender difference in the CO baseline values, *t*(78) = 2.10, *p* = .039, η^2^ = .05, due to higher values for men (*M* = 5.84, *SE* = 0.26) than for women (*M* = 5.26, *SE* = 0.14). No gender difference emerged for the TPR baselines (*p* = .428).

**1.2 CO and TPR Reactivity**

We created reactivity scores by subtracting the baseline values from the averaged 1-min scores of CO and TPR assessed during the task (Cronbach’s αs > .88). Means and standard errors appear in Supplementary Table 2.

| Supplementary Table 3  *Means and Standard Errors (in Parentheses) of Cardiac Output and Total Peripheral Resistance Reactivity (Experiment 1).* | | |
| --- | --- | --- |
|  | *Incongruent Stroop Primes* | *Congruent Stroop Primes* |
| CO | 0.07 (0.04) | 0.11 (0.04) |
| TPR | 16.42 (9.52) | 23.72 (11.37) |
| Notes. CO = cardiac output (in liters per minute), TPR = total peripheral resistance (in dynes second per centimeter to the 5^th^ power). | | |

Preliminary ANCOVAs found no significant associations between baseline and reactivity scores of CO or TPR (*F*s < 0.02, *p*s > .85). As main analyses, independent samples *t*-tests of CO and TPR reactivity revealed no significant effects of the Stroop Primes (*t*s < 0.83, *p*s > .411). Moreover, there was a gender difference in TPR reactivity, *t*(78) = 3.09, *p* = .003, η^2^ = .11, due to higher values for men (*M* = 52.71, *SE* = 10.55) than for women (*M* = 5.82, *SE* = 8.84). No significant gender difference was found for CO reactivity (*p* = .130).

**2. Experiment 2**

| Supplementary Table 4 | | | | | |
| --- | --- | --- | --- | --- | --- |
| *Means and Standard Errors (in Parentheses) of Demographic data (Experiment 2).* | | | | | |
|  | *Assigned characteristics* | |  | *Self-Chosen characteristics* | |
|  | *Incongruent Stroop primes* | *Congruent Stroop primes* |  | *Incongruent Stroop primes* | *Congruent Stroop primes* |
| Gender | 27 women / 6 men | 26 women / 5 men |  | 27 women / 6 men | 28 women / 6 men |
| Age | 23.52 (1.38) | 20.94 (0.63) |  | 20.82 (0.32) | 21.76 (0.91) |
| BMI | 21.46 (0.50) | 20.70 (0.51) |  | 22.73 (0.94) | 23.16 (0.85) |
| Note. BMI = body mass index; Med car = drugs that influence cardiovascular system; Med dep = antidepressant drugs | | | | | |

**2.1 CO and TPR Baseline Values** We constituted CO and TPR baseline scores by averaging cardiovascular values of the last 3 minutes of the habituation period, which showed high internal consistency (Cronbach’s αs > .99). Means and standard errors appear in Supplementary Table 5.

| Supplementary Table 5 | | | | | |
| --- | --- | --- | --- | --- | --- |
| *Means and Standard Errors (in Parentheses) of Baseline Scores (Experiment 2).* | | | | | |
|  | *Assigned characteristics* | |  | *Self-Chosen characteristics* | |
|  | *Incongruent Stroop primes* | *Congruent Stroop primes* |  | *Incongruent Stroop primes* | *Congruent Stroop primes* |
| CO | 5.63 (0.18) | 5.49 (0.19) |  | 5.98 (0.22) | 5.70 (0.20) |
| TPR | 1109.50 (32.93) | 1098.58 (34.69) |  | 1036.48 (33.02) | 1095.58 (44.94) |
| Notes. CO = cardiac output (in liters per minute), TPR = total peripheral resistance (in dynes second per centimeter to the 5^th^ power). | | | | | |

Preliminary 2 (Stroop Prime) x 2 (Choice) ANOVAs found no significant a priori differences between the conditions (*F*s < 2.02, *p*s > .157). Additional *t*-tests found a gender difference in the CO baseline values, *t*(117) = 2.01, *p* = .047, η^2^ = .03, reflecting higher values for men (*M* = 6.16, *SE* = 0.22) than for women (*M* = 5.63, *SE* = 0.11). There was no significant gender difference in TPR baselines values (*p* = .382).

**2.2 CO and TPR Reactivity**

We created reactivity scores by subtracting the baseline values from the averaged 1-min scores of CO and TPR assessed during the task (Cronbach’s αs > .92). Means and standard errors appear in Supplementary Table 6.

Preliminary ANCOVAs found a marginal and a significant association between baseline and reactivity scores of CO and TPR—*F*(1,115) = 3.76, *p* = .055, η^2^ = .03, and *F*(1,115) = 9.60, *p* = .002, η^2^ = .08, respectively. Moreover, there was a significant interaction effect on baseline-adjusted CO reactivity, *F*(1,115) = 6.03, *p* = .016, η^2^ = .05, in absence of significant main effects (*F*s < 1.02, *p*s > .312). However, additional post hoc cell comparisons with HSD Tukey tests revealed no significant differences between conditions (*p*s > .094).

| Supplementary Table 6 | | | | | |
| --- | --- | --- | --- | --- | --- |
| *Means and Standard Errors (in Parentheses) of Cardiac Output and Total Peripheral Resistance Reactivity (Experiment 2).* | | | | | |
|  | *Assigned characteristics* | |  | *Self-Chosen characteristics* | |
|  | *Incongruent Stroop primes* | *Congruent Stroop primes* |  | *Incongruent Stroop primes* | *Congruent Stroop primes* |
| CO | 0.17 (0.06) | 0.05 (0.06) |  | 0.08 (0.06) | 0.25 (0.06) |
| TPR | 7.88 (10.17) | 26.14 (11.43) |  | 26.48 (10.22) | -6.04 (11.02) |
| Notes. CO = cardiac output (in liters per minute), TPR = total peripheral resistance (in dynes second per centimeter to the 5^th^ power).  The reactivity scores are baseline-adjusted. | | | | | |

Also on baseline-adjusted TPR reactivity, there was a significant interaction effect, *F*(1,115) = 5.59, *p* = .020, η^2^ = .05, without significant main effects (*F*s < 0.45, *p*s > .507). HSD Tukey tests revealed no significant differences between conditions (*p*s > .135).

We finally also run the 3:1 a priori contrast that tested our predicted effort-related pattern. That contrast was neither significant for baseline-adjusted CO (*F* = 0.34, *p* = .554) nor for baseline-adjusted TPR (*F* = 0.42, *p* = .521).

Regarding gender differences, men showed stronger CO reactivity (*M* = 0.32, *SE* = 0.11) than women (*M* = 0.10, *SE* = 0.03), *t*(117) = 2.78, *p* = .004, η^2^ = .06. There was no significant gender difference for TPR reactivity (*p* = .464).

**Supplementary References**

Bernstein, D. P. (1986). A new stroke volume equation for thoracic electrical bioimpedance: Theory and rationale. *Critical Care Medicine*, *14*, 904–909. https://doi.org/10.1097/00003246-198610000-00017

Sherwood, A., Allen, M. T., Fahrenberg, J., Kelsey, R. M., Lovallo, W. R., & Doornen, L. J. P. (1990). Methodological Guidelines for Impedance Cardiography. *Psychophysiology*, *27*, 1–23. https://doi.org/10.1111/j.1469-8986.1990.tb02171.x
